# Supplementary material for: Don’t stop me now: Psychological effects of interrupting a moving pedestrian crowd and a video game
Source: PLoS One. 2023 Jul 14;18(7):e0287583. doi: 10.1371/journal.pone.0287583 (PMC10348534; doi:10.1371/journal.pone.0287583)
Supplement: Attachment — Submitted filename: Recommendation.docx [file pone.0287583.s001.docx]

This manuscript focuses on studying the effects of interruptions on individuals from a psychological and crowd dynamics perspective, which is interesting and practical. Experiments and numerical analysis of the results are presented in a very clear way. I would like to make some observation about the work.

1) Abstract should be improved to provide a better overview of the study.
2) Whether the heterogeneity of the research sample (Germany and Turkey) will affect the research results, have you compared the differences of results between the two different countries in study 1?
3) The reviewer advised that the diagram should be put in the text rather than appendix, and the alignment of manuscript is unsightly, please modify the whole manuscript.
4) In line 212, the author mentioned that “The raw data of each participant were then cut manually into their respective time periods”. Why did use to manual way? What is the criterion for cutting?
5) In line 418, the author mentioned that “20 participants from each group (a total of 60) were selected and instructed to wear the device before the experiment began”. Why did only select 20 participants? What is the criterion for selecting 20 participants?
6) The Fig. 3 not present clear information, the reviewer advised to draw the picture for convenience of expressing content.
7) The language of the manuscript should be polished carefully. There are a lot of mistakes inside.
