# Supplementary material for: Don’t stop me now: Psychological effects of interrupting a moving pedestrian crowd and a video game
Source: PLoS One. 2023 Jul 14;18(7):e0287583. doi: 10.1371/journal.pone.0287583 (PMC10348534; doi:10.1371/journal.pone.0287583)
Supplement: Attachment — Submitted filename: Response to Reviewers.docx [file pone.0287583.s002.docx]

**General information**

- We have thoroughly proofread the entire manuscript again, this time with the help of an AI tool (ChatGPT).
- Note that the funding number for Study 2 has been updated to FKZ 13N14531, reflecting a correction from the original manuscript.

**Additional requirements**

- The style requirements have been checked again.
- The data from Study 2 will be uploaded here once the corresponding paper for the full project experiments is published: <https://ped.fz-juelich.de/db/doku.php?id=croma> (preprint: <https://arxiv.org/abs/2303.02319> – accepted for publication, currently in revision phase)
- Ethic statements have been edited for both Studies.

Individual responses to each point raised by the reviewers are provided below.

**Reviewer 1**

1) Abstract should be improved to provide a better overview of the study.

I have improved the abstract to make it clearer and to highlight the notable outcomes.

2) Whether the heterogeneity of the research sample (Germany and Turkey) will affect the research results, have you compared the differences of results between the two different countries in study 1?

The data from the different location samples showed no significant difference from each other, as indicated in the old lines 339-341 (page 15 – Study 1 - Discussion). However, the overall heart rate was higher in the participants in Turkey location, which was presumably caused by the summer temperatures (approximately 10 degrees higher than in Germany [old lines: 217-225]). Nevertheless, the direction of the data was the same in both samples (i.e., no increase during the interruption period).

3) The reviewer advised that the diagram should be put in the text rather than appendix, and the alignment of manuscript is unsightly, please modify the whole manuscript.

All figures have been uploaded separately as per the submission guidelines of the journal, which specifically required that they not be included in the manuscript. However, the alignment of all figures has been edited to be accurate.

4) In line 212, the author mentioned that “The raw data of each participant were then cut manually into their respective time periods”. Why did use to manual way? What is the criterion for cutting?

The individual raw data consisted of all time periods starting from when the device was switched on until the data was exported. This time period of approximately two hours included the experiment time period and the interruption time period. Unfortunately, we were unable to control the device remotely for starting and ending the record since it is not a feature of EcgMove 4. As a result, we had to manually cut the data afterwards. We wrote down all time points indicating the start of the interruption manually (without considering milliseconds) and used these time points to cut the raw data into "interruption time," "game playing time," "all experiment," and so on. There were no criteria for cutting the data apart from the time marks that were recorded during the experiment.

However, as Reviewer 1 pointed out, the explanation provided in line 212 does not fully describe the overall procedure. Therefore, I have updated this section accordingly.

5) In line 418, the author mentioned that “20 participants from each group (a total of 60) were selected and instructed to wear the device before the experiment began”. Why did only select 20 participants? What is the criterion for selecting 20 participants?

Unfortunately, we had a maximum of 20 devices at our disposal. While we briefly mentioned this in the "Limitations" section (old lines: 786-787), I have now added this information to the "Study 2 – Method" section as well.

6) The Fig. 3 not present clear information, the reviewer advised to draw the picture for convenience of expressing content.

Figure 3 seems somehow blurred in the submitted pdf file. Does the reviewer 1 refer to this? However, I have updated the image with more information, and the sequences are now more clearly separated.

7) The lanuage of the manuscript should be polished carefully. There are a lot of mistakes inside.

The manuscript has been proofread again.

**Reviewer 2**

1) Line 128: Is “distortive” what you want to say. Makes sense, but maybe “disrupting” was what you meant. Not sure; please check.

The word “distortive” is changed to “disrupting”.

2) Line 161: “being nearly at the would” --> “being nearly at the end”

Updated accordingly.

3) Line 209: I might be wrong but the fact that participants had to play a game is discussed here taking for granted that readers would know that. But I believe the fact that participants were playing a game is only introduced later (except for the abstract).

In the last paragraph of the "Study 1 - Introduction" section, we explained that due to Covid-19 restrictions, we had to change the experiment setting to a game-playing context (old lines: 165-168). While this information was not included in the introduction(s) apart from here and the abstract, it was a deliberate choice to focus on pedestrian dynamics as the main context of our study. I would like to continue with this background, but I also ensure that the game-playing aspect is properly emphasized.

4) Line 250: I was wondering; why didn’t you ask to fill the questionnaire during the interruption? I am not criticizing the procedure, it is perfect. Personally, I would have asked them to start filling the questionnaire while you “fix the technical” issue, just to have an idea on how they felt. Then you can ask them again to fill the questionnaire at the end. Of course the questionnaire during the interruption need to be a bit different than the final one, but with some deception you may gain a better insight on their state of mind during the interruption. Maybe it could be an idea for a follow-up experiment; again, not criticizing the work.

The main aim was to induce annoyance from "waiting" in participants, so we needed them to wait for at least a little bit to create boredom or impatience, ideally for 10 minutes. If we had given them the questionnaires directly, they would have only experienced annoyance from "not being able to play" but not from "waiting". However, I can see that this is an interpretation difference of pedestrian activity interruption, whether the annoyance of interruption comes from "waiting" or "not being able to proceed," or both. This is really interesting to think about and definitely an idea for a follow-up experiment.

5) Line 262: “around ten participants”; maybe you can provide the percentage, just to have a better idea although the total number is given.

Percentage added.

6) Lines 373-379: Maybe you can add something like “details will follow” just to let readers know that this aspect will be discussed in detail later.

Additional note added.

7) Lines 393-394: I guess you had homosexual, transgender or people not providing their gender (unidentified). Maybe you can add the percentage for “other” just to show that it comes to 100% and there is no mistake in reporting.

Percentage of “other” category added.

8) Lines 396-397: “without” is repeated.

The second “without” is changed to “with”.

9) Line 414: I think you did have a deception because people did not know that the interruption was the goal of the experiment itself. So, I am not sure what you mean here by saying “no deceptive items were used.”

In Study 1, we included many unrelated questionnaire items about positive emotions to avoid making the true purpose of the experiment too obvious to the participants. If we had only included impatience and boredom-related items, it would have been too transparent. However, in Study 2, due to time constraints and rapid experimental structure, we were only able to present impatience and boredom-related items. Although we still used deception, we did not include any deceptive questionnaire items as in Study 1. However, to make this clearer, I have edited the relevant section now.

10) Fig. 3 looks black and white to me although it is stated that orange is used.

I think there was something wrong with my uploads and their quality as Reviewer 1 also pointed out. All images are now re-uploaded. The orange dot (representing the experimenter) should be visible.

11) Fig. 10 was quite unclear to me at first. I would use a different color for the orange in the middle. Using the same color is a bit confusing. Also, maybe you can add the condition (motivation) as a text in the images instead of having it written in the caption. Having some text in the white space of the figure would help reading them. Also, I guess having seconds instead of frames is better (although you wrote that 1 s = 50 frames).

The motivation condition has been added to the plots, and the time frames have been changed to seconds. Although we discussed the color representation, we decided to keep it as is since both time periods represent when the gate is opened and people were allowed to proceed (as before and after the interruption). I also revised the figure caption to provide more clarity.

12) Fig. 11, similar remarks on labeling and x-axis.

Changed accordingly.

13) Lines 817-819: I would be a bit careful in suggesting to stop a crowd considering accidents which occurred in the past. If a crowd has to be stopped the whole crowd must be informed. Having only a small part of the crowd stop moving with the rest in movement can be dangerous in some situations.

Reviewer 2 is right; this part is really important. The aspect also has been added and explained in the respective paragraph now.

14) Graphs: I would better change them a bit just to avoid them being in the standard Excel format. I am not at all against using Excel which is a valid software for visualizing results, but having a choice of color which is not the standard one could help presenting the study in a more “professional” way. Just my personal advice anyway, there is no problem at all in the way it is presented now.

To be honest, I wasn’t happy with the excel exports as well… So, I decided to follow the suggestion, now all images are edited again using Photoshop.
